# Adaptivity of End Effector Motor Control Under Different Sensory Conditions: Experiments With Humans in Virtual Reality and Robotic Applications

**DOI:** 10.3389/frobt.2019.00063

**Published:** 2019-07-24

**Authors:** Jaime Leonardo Maldonado Cañón, Thorsten Kluss, Christoph Zetzsche

**Affiliations:** Cognitive Neuroinformatics, University of Bremen, Bremen, Germany

**Keywords:** contact velocity, motor control, motor learning, robotic learning, hidden Markov models, manipulation task, multisensory, movement with risk

## Abstract

The investigation of human perception and movement kinematics during manipulation tasks provides insights that can be applied in the design of robotic systems in order to perform human-like manipulations in different contexts and with different performance requirements. In this paper we investigate control in a motor task, in which a tool is moved vertically until it touches a support surface. We evaluate how acoustic and haptic sensory information generated at the moment of contact modulates the kinematic parameters of the movement. Experimental results show differences in the achieved motor control precision and adaptation rate across conditions. We describe how the experimental results can be used in robotics applications in the fields of unsupervised learning, supervised learning from human demonstrators and teleoperations.

## 1. Introduction

A key challenge for future robotics is the mastering of everyday activities (Beetz et al., 2015). When entering into everyday life situations, e.g., in kitchens, house cleaning, or in interactions with humans, robots are confronted with a huge variety of objects, material properties, and with a continuously changing environment, in which novel objects never experienced before can show up at any time. The ability to learn and to adapt to the respective object properties is a key feature in such a situation, in particular for the successful manipulation of objects and tool usage. The objects and materials involved may have various properties, for example being easily breakable, like expensive wine glasses, or they may be deformable, like dough, or elastic, like plastic bottles. This implies that a lot can go wrong, if an agent does not apply the appropriate forces, for example. But how can a robot, or a human, learn that something is breakable, cuttable, or deformable? How can one learn, for example, which movement of a spoon is appropriate to just crack the egg-shell, and not leaving it intact, or smashing the whole egg? A key phase in tool-object and object-object interaction is the contact event. Contact events generate characteristic haptic, visual, and auditory information that can help in learning how to deal with tools and objects. Since humans have a mastery in dealing with such everyday situations that is not yet reached by their robotic counterparts, this prompted us to investigate how humans can learn and adapt their behavior on the basis of contact events. In particular, we measured how subjects can learn to control the movement of a tool (spoon) toward an object with a desired contact force, based on haptic and auditory information about the contact event.

The contribution of individual sensory channels to the human sensorimotor loop is difficult to investigate since sensory events are usually experienced as one multisensory unit in the natural world. Therefore we used a virtual reality experimental setup augmented with haptic rendering and real-time sound synthesis, which enabled us to present participants with separate sensory information during a motor task. With the aim of using the acoustic information generated during manipulation tasks to improve the robot's capabilities, we investigate human kinematic control under different sensory conditions. In particular, we aim to model the extent to which humans can use auditory information to adapt sensory motor patterns.

The goal of the analysis of kinematic control is to model how humans learn to master motor tasks, how tasks execution is adapted to different requirements and how the learning and adaptation processes are enabled by the available sensory information. In particular, models of motor control, learning and adaptivity under different sensory conditions can be used to determine the extent to which one sensor modality can be replaced by others in the case of missing, noisy, or incomplete sensory feedback.

In the present study, we investigate the influence of sensory feedback on human movement behavior during a contact event. This work provides evidence on how the sensorimotor system performs control actions during motor tasks given the afferent information available. Motor control and task performance are investigated in terms of the kinematic parameters of the movement and their adaptation due to the repetition of the task and the available sensory information. The experimental results illustrate the individual contribution of haptic and auditory information to task performance as well as the strategy used by the motor control system triggered when haptic information is missing. Based on the significant differences found across conditions and blocks of trials we quantified the rate at which the movement parameters adapt in each condition. Furthermore, we describe three areas of application in robotics in which our experimental results can be applied: unsupervised learning, supervised learning from data and human demonstrators, and teleoperations.

## 2. Related Work and Background

During object manipulation tasks, action phases are generally delineated by distinct sensory events that represent task subgoals. The motor control system uses afferent signals and sensory predictions to modulate motor output according to the requirements imposed by the task (Johansson and Flanagan, 2009).

During the events that mark transitions between action phases, such as the moment of contact of a tool with a surface, the brain receives sensory information from multiple sources; visual, haptic, and auditory modalities encode information about the physical nature of the contact event (e.g., force and contact duration) as well as information related to object properties (e.g., size, shape, material) (Cook, 1997; Van Den Doel and Pai, 2007; Johansson and Flanagan, 2009). These multisensory representations enable humans to learn sensorimotor correlations that can be used to monitor task progression and to trigger corrective actions if mismatches between the predicted and the actual sensory outcomes are detected (Johansson and Flanagan, 2009).

Models based on human manipulation have been used as a basis for the design of robotic systems (for a review see Yousef et al., 2011). Manipulation consists of a sequence of action phases separated by contact events, each fulfilling a subtask (Johansson and Flanagan, 2009; Yousef et al., 2011). A prototypical manipulation task consists of reach, load, lift, hold, replace, and unload phases (Johansson and Flanagan, 2009). Contact events correspond to sensory events in the haptic, visual, and auditory modalities. The goal of the unload phase is marked by the moment in which the object touches the support surface, and is characterized by the contact force and the impact sound. The detection of the force and the magnitude of contact forces due to the motion of the hand during manipulation is regarded as one of the minimum functional requirements for a robotic sensing system mimicking human in-hand manipulation (Yousef et al., 2011). This paper focuses on this functional requirement by investigating the control of force during the contact phase of a prototypical manipulation task.

In the field of robotics different sensors can measure physical quantities during task performance. The fusion of information coming from different sensor modalities is used for environment perception, system diagnosis, and decision making (Luo and Chang, 2012). The manipulation capabilities of mobile robots are regarded as weak compared with the motion and sensing abilities, due to the immature cooperation between the sensor-motor systems (Luo and Chang, 2012). A key feature of human manipulation is the ability to perceive the consequences of an action. Robots are still missing this ability, as exemplified by Stelter et al. (2018). Force sensing in robotic systems, which is essential for skillful manipulations, is limited when compared to human capabilities. Robotic systems can overcome the limitations of force sensing by exploiting the acoustic information generated by tool-object, or actuator-object interactions, improving the robot's capabilities to extract information about the materials (e.g., texture or weight), the kinematics of an interaction (e.g., contact tool-object contact velocity) and the monitoring of task progress.

Robots operating in changing environments or under uncertain conditions must adapt constantly to the new circumstances imposed by the context. Furthermore, uncertain conditions might also arise during the execution of well known tasks (e.g., a robot manipulating a liquid container, in which the amount of fluid can't be specified a priori). Therefore, models of human adaptivity can inspire the design of adaptive robotic systems.

The sensory information generated during the manipulation of an object informs not only about the success of the contact event but also about its quality in form of the haptic sensation and, most prominently, the acoustic sensation. This so called impact sound is characterized by its short duration, an abrupt onset and a rapid decay (Aramaki and Kronland-Martinet, 2006). It encodes perceptual information about the physical attributes of the object (material, shape, size) and the strike movement [force of the impact and the location of the collision on the object's surface (Cook, 1997; Van Den Doel and Pai, 2007)]. Information encoded in impact sounds highlights their importance for the performance of manipulation tasks, especially when human-like precision is required.

Information encoded in sound has been used in robotics for the perception of events and the recognition of objects (Sinapov et al., 2011; Luo et al., 2017b). Information encoded in sound has also been used in the recognition of materials (for a review see Luo et al., 2017a). In these applications, the characteristic features of the sound produced by an action (i.e., the acoustic signature from an action-object interaction) are used in classification tasks. Actions performed by robotic actuators that produce an acoustic signature include tapping, lifting, shaking, dropping, crushing, and pushing (Sinapov et al., 2011). Thus far, robotic systems have mainly used the information about object material, shape and size encoded in acoustic signals, setting aside information about the event that generated the sound.

With respect to the haptic component, contact forces play a major role in tool manipulation. A contact force involves the actions exerted by one object in direct contact with another (Hamill et al., 2015). Contact forces are essential to the execution of everyday activities in which tools act on objects. These forces are perceived as distinct sensory events that represent action task subgoals (e.g., initial tool-object contact). These afferent signals are used by the motor control system to modulate the motor output according to requirements imposed by the task (Johansson and Flanagan, 2009).

All surfaces and objects on which an individual interacts provide a reaction force. During the impact phase between two objects the force increases with increasing indentation while the velocity at which the objects are approaching each other is reduced (Machado et al., 2012). When a hand-held tool makes contact with an object's surface the reaction force is proportional to the acceleration of the tool combined with the hand and arm forces. The afferent signal generated by the reaction force indicates the person that contact has taken place. Once tool and object are in contact, the reaction force is used to control the rest of the interaction (e.g., keep tool in contact with the object at a desired position or with a target force to perform a task). During the contact onset of a hand-held object and a surface the impact force is transmitted to the fingers and the upper arm. Muscular activity observed during collisions of hand-held tools and surfaces may reflect a mixture between anticipatory and purely reactive responses triggered by fast adapting mechanoreceptors (White et al., 2011).

Moreover, voluntary movements of hand-held objects produce predictable modulations of the load (Latash, 2013). In Rapp and Heuer (2015), the effects of the predictability of abrupt offsets of horizontal forces on initial conditions and kinematic characteristics of the movements triggered by the force offset were investigated. Their experimental results show that anticipatory adjustments resulted in shorter peak amplitudes of the involuntary movements triggered by abrupt force changes and that the modulations of movement times depended on the different states of the limb at the moment of change.

Anticipatory adjustments are regarded as an example of the predictive nature of motor control (White et al., 2011). When the abrupt unloading or loading of the hand can be predicted, anticipatory adjustments of muscular activity or changes of limb impedance can be observed (for examples see Rapp and Heuer, 2015). Apart from the adjustments observed in White et al. (2011) and Rapp and Heuer (2013, 2015), anticipatory behavior observed during task performance also includes the velocity decrease when the target is approached in reaching movements. Similar velocity reductions occur in vertical and horizontal movements when the hand or hand-held tool approaches a contact surface.

## 3. Materials and Methods

### 3.1. Participants

Ten volunteers (five females and five males) with an age range of 26–40 years participated in the experiment. Nine participants were right handed. All participants had normal or corrected-to-normal vision and reported having no motoric problems. All subjects gave written informed consent in accordance with the Declaration of Helsinki. The experiment was approved by the ethics committee of the University of Bremen.

### 3.2. Apparatus

Stimulus presentation and control of the experiment was accomplished with Vizard 5 (WorldViz). Haptic stimuli were generated using a Phantom Premium 1.5 (3D Systems) force-feedback device, equipped with a stylus which provides 3 degrees of freedom positional sensing (x, y, and z coordinates), 3 degrees of freedom force-feedback and measurement of pitch, roll and yaw. Interaction with the virtual objects is achieved by manipulating the stylus. Feedback force is rendered at the tip of the stylus when it touches the surface of a virtual object.

The audio was presented through DT 770 (Beyerdynamic) headphones, the sound synthesis was implemented in ChucK (Wang and Cook, 2003). The output from the sound synthesis module was mixed with pink noise. The pink noise was presented continuously in order to mask the sound of the Phantom hardware. Communication between virtual reality software and the sound synthesis module was implemented with the Open Sound Control (OSC) protocol.

**Figure 1:**
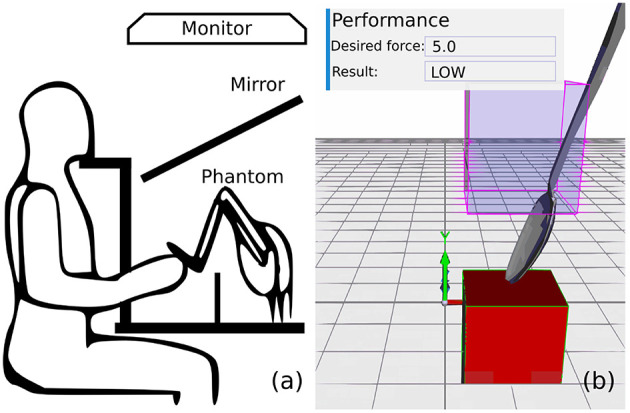
Experimental setup. **(a)** Schematic illustration of the experimental setup. **(b)** 3D virtual environment.

The experimental setup is shown in Figure 1. Participants sat down with their head placed on a chin rest and looked at the image being reflected in a mirror from a monitor (EIZO Flexscan F784-T). The viewing distance to the mirror was 15 cm. The Phantom hardware and the participants' hands were occluded by the mirror.

### 3.3. Stimuli

Participants viewed a 3D environment containing a red box (termed contact box) (15 × 15 × 20 cm) and a spoon (see Figure 1). The visual and haptic stimuli were aligned spatially. The virtual spoon was attached to the stylus of the Phantom; thus the stylus manipulation was reflected in its position and orientation. In addition, a semitransparent box (termed start box herein) was displayed to signal the start position of the movement. The center of the contact box was located at x = 12 cm, y = 10 cm, z = −9 cm from the origin and the center of the start box at x = 12 cm, y = 22 cm, z = −9 cm. The impact sound was synthesized and force was rendered when the tip of the stylus reached upper surface of the contact box. Participants were requested to hold the stylus like they would hold a pen, so that the tip of the spoon was always pointing down.

#### 3.3.1. Sound Synthesis

When a solid object is struck the forces at the contact point cause its outer surfaces to vibrate and emit sound (Van Den Doel and Pai, 2007). In order to investigate impact sounds and their perception, a sound synthesis module was implemented, taking into account the physics of the object and the kinematic characteristics of the movement that caused the impact. In this “physically informed” approach the parameters of the synthesis system have physical meaning (Cook, 1997) aiming to reproduce an impact sound from a perceptual point of view (Aramaki and Kronland-Martinet, 2006).

**Figure 2:**
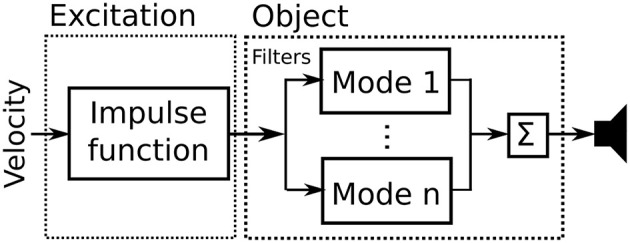
Block diagram of the modal synthesis algorithm.

Impact sounds were generated in real time by a modal synthesis algorithm that enables modeling of the vibrating object and excitation physics (Cook, 1997; Aramaki and Kronland-Martinet, 2006). The synthesis system, shown in Figure 2, consists of an excitation block and an object block. The implementation is closely based on the systems described in Cook (1997) and Van Den Doel and Pai (2007).

In the object block a vibrating object is modeled by a bank of resonant band-pass filters. The model parameters (modal frequencies, decay rates, and the gain coefficients of each mode) were obtained experimentally by recording the impulse response of a glass surface. The synthesis model utilizes 68 modes at 1 contact point. Thus, impact on the virtual object corresponds to a vibrating glass surface.

In the excitation block the force was modeled based on the coupled hammer-object model used by Avanzini and Rocchesso (2001), in which the contact force is proportional to the velocity of the strike. The velocity of the strike was mapped to the amplitude of an impulse function used to excite the filter bank. For the real time interaction, velocity was approximated by the difference between the current discrete time position and the last, divided by the time step.

### 3.4. Experimental Task

The participants' task was to move the spoon away from the start position until it touches the contact box, and they were requested to try to do this with a target force. The target force was displayed as a dimensionless force score on the top of the screen (see Figure 1), which reflects the proportionality of the velocity of the strike and the contact force (*score* = 10[*s*/*m*] × *velocity*[*m*/*s*]). The target force score was set to 5, which corresponds to 0.5 *m*/*s*, and it was constant throughout the experiment and across participants.

At the beginning of each trial, participants moved the spoon toward the start box. Participants were instructed to hold the spoon within the start box. Once a steady position was detected, the start box changed its color from red to green, resembling a traffic light. Participants were requested to hold the spoon steady until the box turned green and then to move it vertically in order to touch the contact object. The trial ended at the moment of contact.

At the end of the trial, the force error was computed as *error* = *score* − *target*. The feedback about the contact force was given with three linguistic labels displayed in the result text box on the top of the screen. Force was described as *LOW* when *error* < −2, *GOOD* when *abs*(*error*) ≤ 2, and *HIGH* when *error* > 2. After the feedback was displayed, the start box changed its color to blue and participants were instructed to proceed with the next trial. Participants were explicitly instructed to achieve an accurate contact force across trials in order to maintain *GOOD* performance throughout the experiment.

There were three experimental conditions that differed in the sensory information delivered at the moment of contact between the spoon and the contact object. In the haptic condition (H) force was rendered when the tip of the spoon touched the surface of the contact box. In the acoustic (A) condition impact sound was synthesized when it reached the surface of the contact box. In the haptic-acoustic condition (HA) force was rendered and audio was generated. Visual information was available in all the conditions, thus they only differed on the availability of haptic and acoustic information.

After the initial instruction, participants were allowed to do practice trials in order to get used to the experimental setup. Participants performed the experiment in 3 blocks of 50 trials (one block per condition). The order of the conditions was randomized. At the end of each block participants took a short break. Participants were instructed to report arm fatigue at any time during the experiment.

### 3.5. Data Analysis

Position data from the Phantom were sampled at 60 Hz and preprocessed using a 4th order two-way low-pass Butterworth filter. The cutoff frequency was determined for each participant using residual analysis (Yu et al., 1999; Winter, 2009). Position data were subsequently differentiated to calculate the absolute velocity.

Kinematic features were computed from the position and velocity data. The peak velocity (PV) and average velocity (AV) of the movement as well as the percentage of time to peak velocity (TPVP), which expresses the acceleration time in relation to the complete movement time, were calculated to describe the characteristics of the movement.

**Figure 3:**
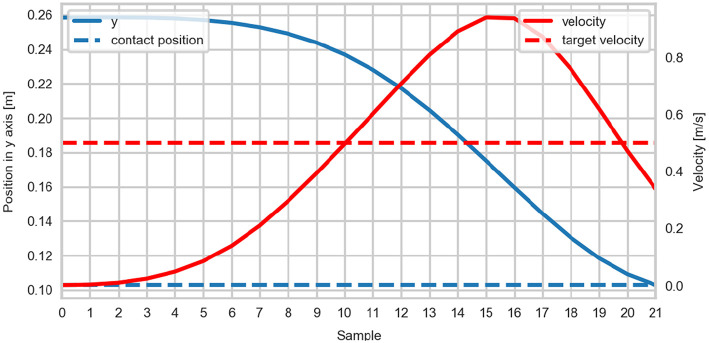
Representative vertical position and velocity curves during a trial.

The relation between the magnitude of PV to the contact velocity (CV) was quantified as the ratio of contact to peak velocity (CTP). The agreement between the CV and the target contact velocity was assessed by calculating the percentage deviation (DEV) computed as *abs*(*CV*−*targetvelocity*)/*targetvelocity*. An example of the position and velocity curves during a trial is shown in Figure 3.

## 4. Results

A two-way repeated measures MANOVA (α = 0.5) with the experimental condition and the trial block (one block representing five consecutive trials) and CV, CTP, AV, PV, TPVP, and DEV as dependent variables shows significant main effects of the experimental condition, $Wilks^{\prime }\Lambda =0.11;{\;\text{F}}_{\left(12,186\right)}=30.1;\;\text{p}<0.001$, and trial block, $Wilks^{\prime }\Lambda =0.77;{\;\text{F}}_{\left(54,2,228\right)}=2.1;\;\text{p}<0.001$. This result indicates a difference of at least one of the variables PV, AV, and TPVP between conditions and in the course of the trial sequences. A significant interaction between factors, $Wilks^{\prime }\Lambda =0.79;{\;\text{F}}_{\left(108,5,292\right)}=2;\;\text{p}\;<\;0.001$, indicates that the kinematic characteristics in the course of the trial sequences are themselves modulated by sensory feedback. As a second step, a closer examination using individual ANOVAS was performed: CV data show significant main effects of the experimental condition, *F*_(1.2, 58)_ = 185.8; *p* < 0.001, trial block *F*_(4.5, 221)_ = 3.5; *p* < 0.01, and a significant interaction between factors, *F*_(7.9, 386.6)_ = 3; *p* = 0.03. AV data show significant main effects of the experimental condition, *F*_(1.6, 80)_ = 80; *p* < 0.001, trial block *F*_(4.5, 221)_ = 4.5; *p* < 0.01, and a significant interaction between factors *F*_(5.8, 283.3)_ = 3; *p* = 0.02. The same applies to PV data, which show significant main effects of the experimental condition, *F*_(1.7, 84.3)_ = 52.1; *p* < 0.001, trial block *F*_(3.6, 176)_ = 5.1; *p* = 0.001, and a significant interaction between factors *F*_(7.7, 487)_ = 2.5; *p* = 0.01. Interestingly, CTP data show a significant main effect only of the experimental condition, *F*_(1.1, 55.7)_ = 191.5; *p* < 0.001, but no effect of trial block *F*_(6.1, 300)_ = 1.6; *p* = 0.14. Furthermore, DEV data rather show a trend but no significant main effect of the experimental condition, *F*_(1, 52)_ = 3.8; *p* = 0.056, and a significant main effect of trial block *F*_(5.8, 284)_ = 2.3; *p* = 0.034. Finally TPVP does not differ significantly, neither between experimental conditions, *F*_(1.9, 91)_ = 2.7; *p* = *ns*, nor between trial blocks, *F*_(4.5, 218.8)_ = 0.87; *p* = *ns*.

In summary, the inferential statistics support, that the differences of movement behavior between sensory conditions and changes of movement behavior over the course of time (i.e., trials), which may reflect motor learning, are evident in the data of the kinematic parameters CV, PV, AV, whereas CTP only expresses the influence of sensory conditions but no change over time. DEV data are not affected by sensory feedback (it may be considered as minimally affected concerning the controversial issue whether near significant results indicate systematic trends as e.g., Wood et al., 2014 discuss) but there are significant changes in the course of time. TVPV data does not reflect any systematic influence at all, neither of sensory feedback nor over the course of time.

*Post-hoc* pairwise comparisons (Bonferroni corrected) of the kinematic parameters that proved significant in the aforementioned analyzes reveal the effect of particular sensory feedback conditions: CV data show a significant difference between the A condition compared to the H (*p* < 0.001) and the HA (*p* < 0.001) conditions, whereas H and HA conditions do not differ significantly (*p* = *ns*). Similar patterns also apply to the kinematic features AV, CTP, PV: there is a significant difference of the A condition in comparison to the H (AV, CTP, PV: *p* < 0.001) and HA condition (AV, CTP, PV: *p* < 0.001), whereas there is none (AV, CTP, PV: *p* = *ns*) between the H and HA conditions.

### 4.1. Contact Velocity

**Figure 4:**
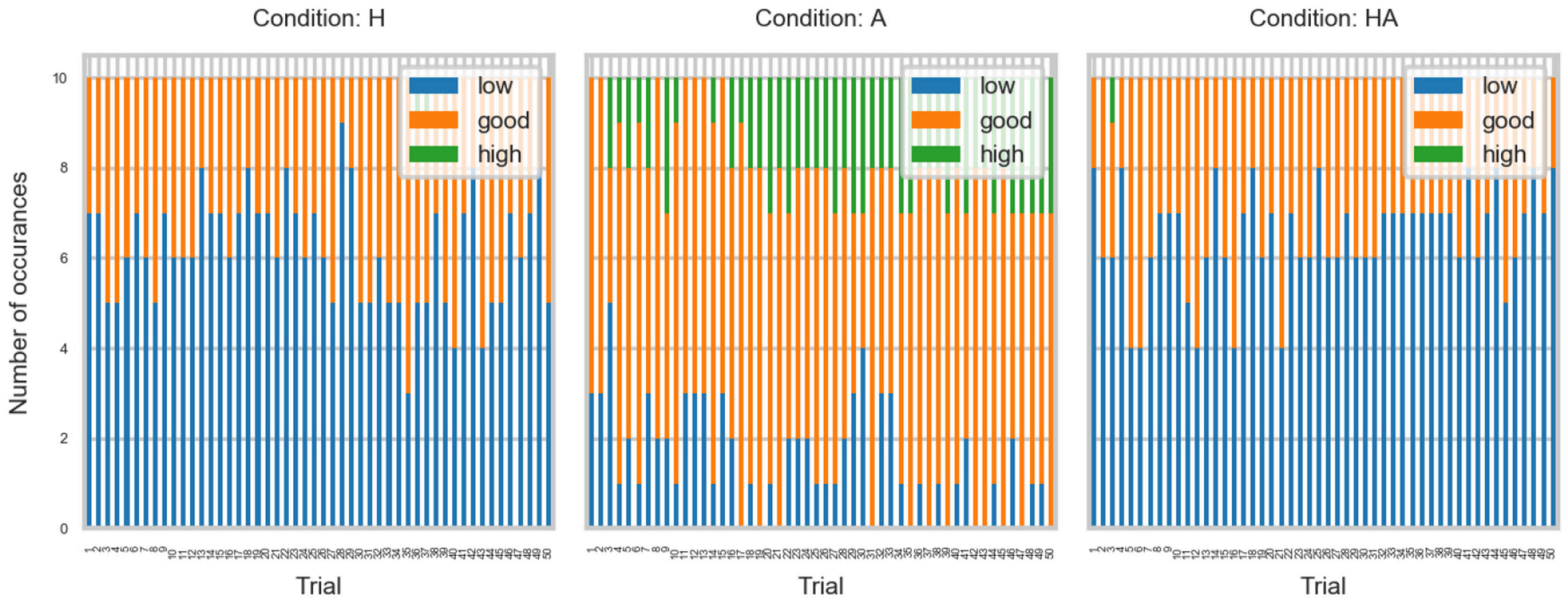
Feedback labels for the haptic (H), acoustic (A), and haptic-acoustic (HA) conditions.

We examined the contact velocity to asses the participants' task performance. Figure 4 shows the qualitative feedback labels (i.e., LOW, GOOD, HIGH) obtained for all participants throughout the experiment. It can be observed that GOOD performance was achieved more often in the A condition, while performance in the haptic conditions tended to be LOW.

**Figure 5:**
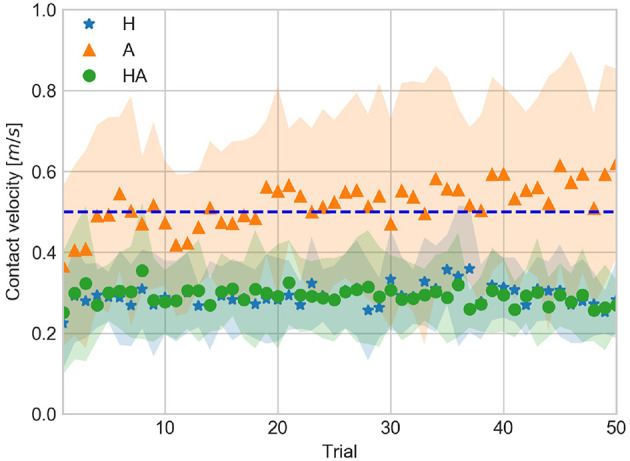
Contact velocity against trial number averaged over all subjects for the haptic (H), acoustic (A), and haptic-acoustic (HA) conditions. The dashed line represents the target contact velocity (0.5 *m*/*s*). The contour represents the standard deviation.

CV data averaged for all subjects across trials are shown in Figure 5. It can be observed that participants were closer to the target velocity in the A condition. Figure 5 also shows the CV was similar in the haptic conditions.

**Figure 6:**
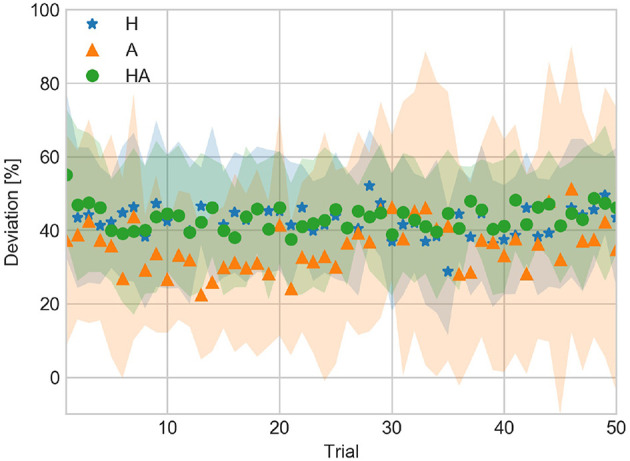
Deviation from contact to target velocity against trial number averaged over all subjects for the haptic (H), acoustic (A), and haptic-acoustic (HA) conditions. The contour represents the standard deviation.

DEV averaged for all subjects across trials is shown in Figure 6. Throughout the experiment DEV tended to be around 40% in the haptic conditions. On the other hand, DEV in the A condition was not uniform throughout the experiment.

**Figure 7:**
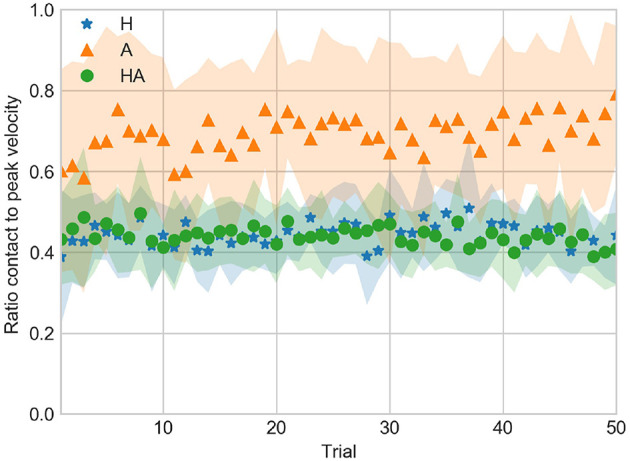
Ratio of contact to peak velocity against trial number averaged over all subjects for the haptic (H), acoustic (A), and haptic-acoustic (HA) conditions. The contour represents the standard deviation.

CTP averaged for all subjects across trials is shown in Figure 7. The CTP tended to be between 0.4 and 0.5 in the haptic conditions. On the other hand CTP tended to be around 0.7 in the A condition. This indicates that the deceleration toward the contact position was larger in the haptic conditions. This suggest that participants developed a different strategy to perform the task in the A condition, in which participants would stop only after the contact sound was generated (due to the lack of a physical limit) instead of the abrupt stop at the contact surface that occurred in the haptic conditions.

CV, DEV, and CTP data indicate that participants used a different control strategy to achieve a GOOD performance throughout the experiment. Thus, the strategy used in the A condition enabled them to approach more often the target velocity. However, this led to a less stable (i.e., less repeatable) performance across trials as illustrated by the DEV data and the larger standard deviation of CV.

### 4.2. Kinematic Features

**Figure 8:**
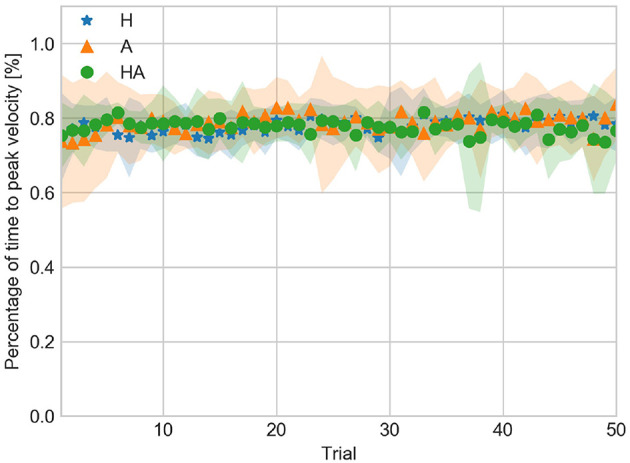
Percentage of time to peak velocity against trial number averaged over all subjects for the haptic (H), acoustic (A), and haptic-acoustic (HA) conditions. The contour represents the standard deviation.

TPVP data are shown in Figure 8. TPVP indicates that the duration of the acceleration and deceleration phases were asymmetric. TPVP data show that the acceleration phase took about 90% of the movement time in all conditions. This result suggests that the timing of events was not affected by the availability of sensory information.

**Figure 9:**
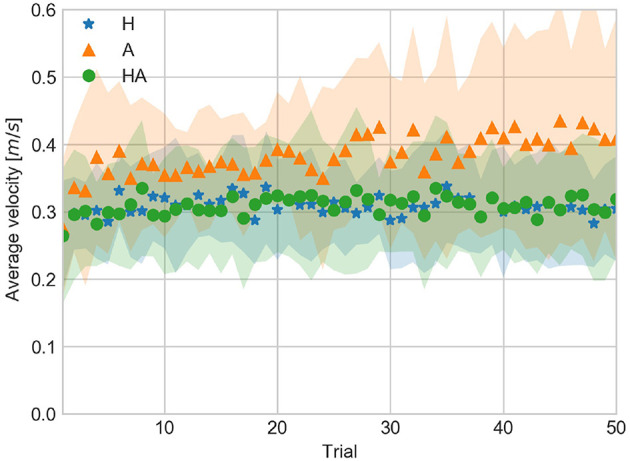
Average velocity against trial number averaged over all subjects for the haptic (H), acoustic (A), and haptic-acoustic (HA) conditions. The contour represents the standard deviation.

**Figure 10:**
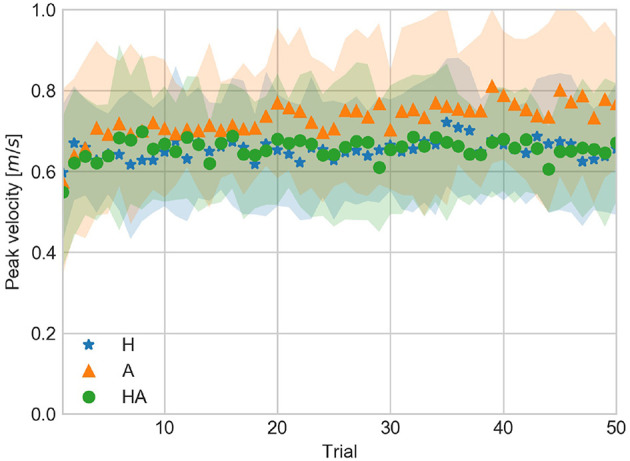
Peak velocity against trial number averaged over all subjects for the haptic (H), acoustic (A), and haptic-acoustic (HA) conditions. The contour represents the standard deviation.

AV and PV are shown in Figures 9, 10, respectively. Similarities in AV and PV between H and HA conditions suggest that motor control is more precise when haptic information is available. By contrast, AV and PV tend to be larger and show a larger variability, as observed in their standard deviation, in the A condition. This suggests that it is more difficult to achieve precise motor control when only acoustic information is available.

The similarities between H and HA conditions suggest that haptic information has a larger influence in motor control. In this case, the motor control system assigns a larger weight to the incoming haptic information. Thus the contribution of the less reliable acoustic information can't be distinguished in the HA condition.

The larger values of AV and PV in the A condition, observed approximately after trial 25, might reflect an active perception strategy which the participants use to increase the reliability of the acoustic information in order to cope with the requirements of the task (i.e., achieve a GOOD contact). Increasing the velocity produced louder contact sounds, making acoustic information more reliable, at the cost of exceeding the task requirement, thus going through the contact surface due to the absence of physical limit. The variability of the AV and PV in the A condition seem to reflect the conflict induced in the motor control when trying to improve acoustic information while coping with the requirement of the task. Further experiments can be devoted to investigate the differences in reliability of haptic and acoustic information.

### 4.3. Adaptation of Kinematic Parameters

The temporal evolution of CV, AV, and PV data is shown in Figures 5, 9, 10, respectively. The significant differences among trial blocks shown in the statistical analysis indicate that the kinematic parameters were modulated by the sensory feedback available in each condition as participants repeated the task. In order to assess the adaptation of the kinematic parameters we fit an exponential function to the CV, AV, and PV data. The *a*, *b*, and *c* parameters of the exponential function (see Equation 1) are interpreted as *leading value, learning rate*, and *asymptote*. This interpretation of the curve parameters is taken from Danion et al. (2013).

(1) $$\begin{array}{r} y=a\cdot e^{-b*x}+c \end{array}$$

**Figure 11:**
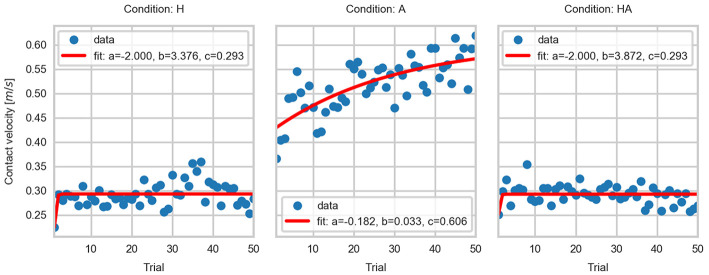
Exponential fits to the contact velocity data.

**Figure 12:**
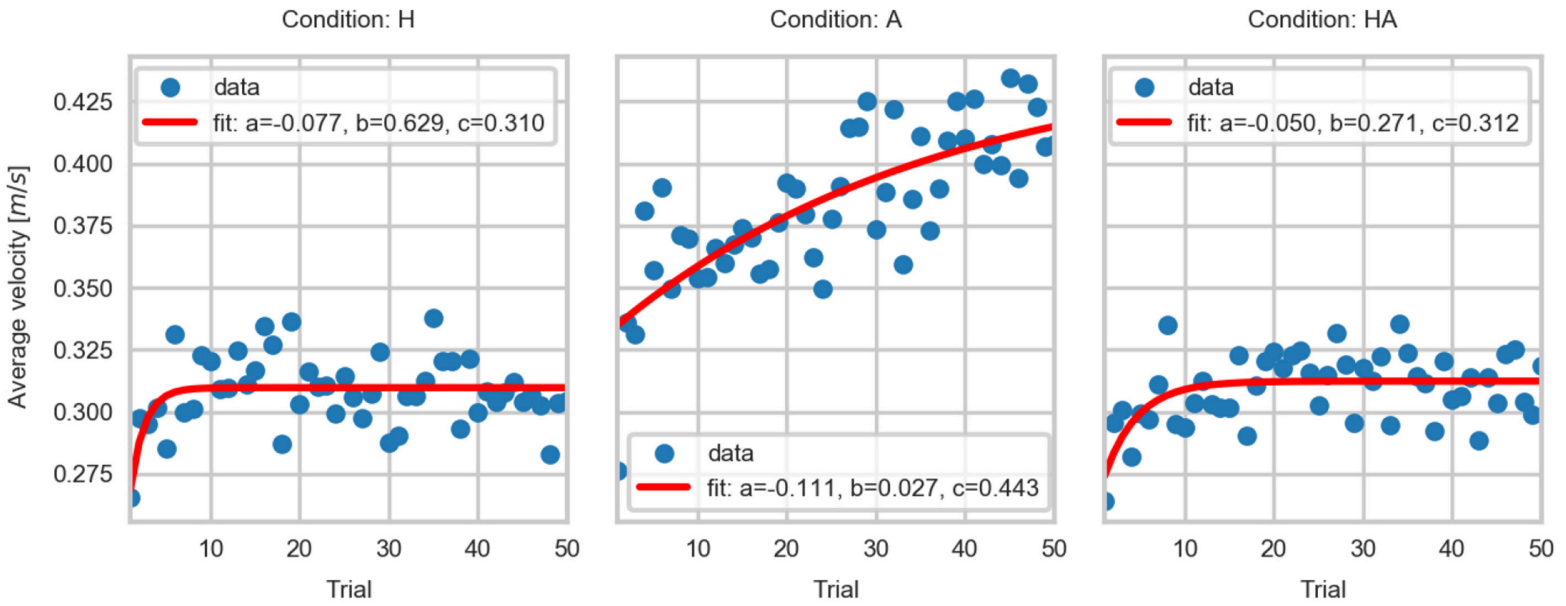
Exponential fits to the average velocity data.

**Figure 13:**
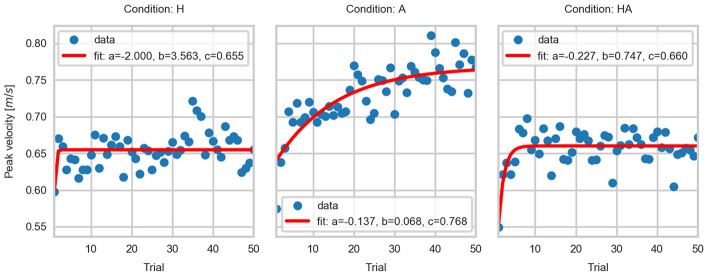
Exponential fits to the peak velocity data.

The parameters of the fitted curves are summarized in Table 1. The fitted curves are shown in Figures 11–13. The curve parameters illustrate the differences in the adaptation of the movement kinematics throughout the experiment. The learning rate was larger in the haptic conditions, thus indicating that movement parameters reached a stable point with fewer repetitions. The asymptotes of the haptic conditions show a similar operating point. In contrast, the fitted curves for the A condition show that a stable point of operation was not achieved. This indicates that the active perception strategy used by participants didn't enable them to achieve a stable operation within the given number of trials. Thus, it would have been necessary a larger number of repetitions in order to achieve a stable and precise task performance.

**Table 1:** Parameters of the fitted exponential curves: (a) leading value, (b) learning rate, and (c) asymptote.

	**CV**			**AV**			**PV**		
	**a**	**b**	**c**	**a**	**b**	**c**	**a**	**b**	**c**
A	−0.182	0.033	0.606	−0.111	0.027	0.443	−0.137	0.068	0.768
H	−2	3.376	0.293	−0.077	0.629	0.310	−2.000	3.563	0.655
HA	−2.000	3.872	0.293	0.050	0.271	0.312	−0.227	0.747	0.660

### 4.4. Discussion of Experimental Findings

Interestingly, the low CV values observed in the H and HA conditions suggest that the sensory feedback provided was impeding participants to increase the velocity regardless of the LOW linguistic feedback provided. In comparison, in the A condition participants exceeded more often the target velocity. In other words, participants were more conservative to try high velocities when haptic feedback was available. This can be regarded as a difference in the motor adaptation process, in which the range of velocities tried out by the motor system to find the target performance differed between the acoustic and the haptic conditions.

We hypothesize that a certain risk is implicit in the haptic conditions, which leads participants to achieve lower velocities. Being too fast in the haptic conditions leads to contacts which might be perceived as harsh or rough, whereas in the A condition the movement can be stopped smoothly after the contact sound has been produced. Thus, apart from the goals of the task, the consequences of increasing the contact velocity are different.

This indicates that the consequences and the risk aspect of the actions need to be explicitly addressed in further investigations of motor control with individual sensor modalities. Feedback provided to participants should not be limited only to the parameters of the action (e.g., contact velocity), but also include information about its consequences (e.g., breaking virtual objects when manipulation velocity exceeds a threshold). Adding any form of explicit feedback about the consequence in the A condition might have led to a different motor adaptation. Thus, we argue that both implicit and explicit feedback about an action's consequences must be addressed, as they might influence task execution and the process of motor adaptation.

## 5. Modeling Human Adaptation of Kinematic Parameters and Task Skills for Robotics Applications

Regarding the categories of robot motion planning proposed by Spiers et al. (2016), *end-effector control* is the most common application of motion planning. In this type of motion planning the motions of the robot gripper or tool are specified in the Cartesian space, as opposed to the use of symbolic instructions (Spiers et al., 2016). Since the end-effector position and its orientation are specified by the programmer, inverse kinematics are employed to control the other joints and links involved in the movement (Spiers et al., 2016).

The experimental task given to the participants, in which the kinematics of a grasped tool were controlled in order to make contact at a target position, resembles the end effector motion planning in robotics. Thus, the models of human motor control and motor adaptivity are suitable of being applied in robotics problems in which the position and the kinematics of end-effector are crucial to achieve a successful and skillful manipulation. In the following subsections we describe how models of human control can be applied to the modeling of action performance regularities for robotics. We distinguish three areas in which our results could be of interest: (i) unsupervised learning, (ii) supervised learning from data and human demonstrators, and (iii) teleoperations.

### 5.1. Unsupervised Learning

A long-term perspective in robotics is that robots can continuously improve and adapt their sensorimotor performance based on their experiences with objects and environments in everyday situations. With respect to the handling of tools and objects, learning the appropriate contact forces is a central requirement. What could be the sources of this learning process? Haptic force measurement is an obvious source but the respective sensor technique is not yet well-established and expensive. Our data indicate that a much simpler source, acoustic information, which requires only microphones and appropriate signal processing, could be used as an alternative for learning appropriate contact forces. Learning may take somewhat longer, and the precision level achieved could be somewhat reduced, but it is possible to adapt the motor system to a desired level of contact force on the sole basis of acoustic information.

It should be noted that even if there is no explicit force measurement, certain aspects related to contact and force which have not been available to our subjects in the VR setup will be available for robots acting in a real-world setting. Even without force measurement the robot movements will be stopped, or discontinuously decelerated, at the moment of contact. This can be sensed both with visual information and with crude readings from the motors. This additional information may well help to improve the precision level beyond the level reached by our subjects (they have only crude information about the time of contact because their movement of hand and spoon is not stopped, but rather the spoon goes undisturbed through the virtual object surface).

A further point that has to be considered in unsupervised learning of robots is the handling of risk. Our subjects have been surprisingly sensitive to risk, but only in the haptic conditions. As described, this might be due to the peculiarities of the VR setup, which encouraged a risky behavior in the A condition. Thus, the different influence of the consequence (or risk) aspects of a task (e.g., if velocity>threshold then object breaks) vs. the simple specification of the task (e.g., keep velocity below a threshold) observed in human motor control and motor adaptation can be used to inspire the design of robotic systems. In general, robots should avoid to destroy things, but too careful behavior could also impede learning. A robot's adaptation or active perception behavior can include certain conditions in which risk is allowed in order to find an optimal motor plan (e.g., when learning to manipulate glasses with different amount of liquid the robot should be allowed to spill some liquid in order to find the optimal manipulation velocity). Risk level setting thus has to be an external decision which takes environmental and learning aspects into account.

### 5.2. Supervised Learning of Action Performance Regularities for Robotics

In the context of interactive perception, Bohg et al. (2017) state that there is a regular relationship between actions and their sensory response, which can be exploited by robotic systems for the prediction and interpretation of these sensory signals. The information necessary to learn the regularity of an action can be also provided by a human demonstrator. In addition to the regularities between actions and their corresponding sensory signals, we propose to inform the robotic system about the regularity of the action performance by a human demonstrator. The regularities of the action performance can be modeled in terms of the statistical properties of the kinematic parameters and the learning curves.

The motions recorded under different sensor modalities can be considered as a series of examples from a set human demonstrators. A robotic system can learn from observing how humans perform a task under different conditions. In particular, the learning curves are useful to estimate how many repetitions are needed by a human demonstrator to achieve a stable (regular) performance under different sensory conditions.

### 5.3. Uni- and Multi-Modal Task Performance for Teleoperation

Modeling of task performance under different sensory conditions can be applied in the field of teleoperation, in particular, when the system can't transmit and reproduce the complete sensory information relevant for the task. This is of particular relevance in teleoperation, since visual and acoustic information is simple to record and to reproduce, while haptic information is already difficult to measure but even more difficult to reproduce. Our evidence that acoustic information can be used as (partial) replacement of haptic information is hence an encouraging result.

In the context of learning from demonstration in telerobotics, teachers or demonstrators must master the task without having the complete sensory information (Billard et al., 2016). Our experimental results model performance differences across different sensory conditions and the leaning curves provide an estimate of the amount of trials needed to achieve a stable point of operation. Furthermore, our experimental results can be generalized as we model the motor control skill across different demonstrators.

Output of real-world processes can be characterized as signals (Rabiner, 1989). The hidden Markov model (HMM) is an stochastic signal model which works based on the assumption that the signal can be characterized as a parametric random process (Rabiner, 1989). A HMM models an stochastic process as set of finite states connected by transitions. This model is characterized by its transition probability matrix (A), an output probability matrix (B) and the initial state distribution (π) (for a tutorial review on HMM models see Rabiner, 1989).

HMMs have been used to represent human skill in the context of telerobotics operation (Yang et al., 1994). Yang et al. (1994) used position and velocity trajectories to learn human skill in the spatial and velocity domains, aiming to select the best task performance from the observed data. The goal of the skill modeling was to obtain the most likely human performance from a set of training examples. In this approach, the HMM represents the most likely performance of a given task. As the task performance depends on the person's skill, the model was used by the robotic application to select actions from the input data.

Following a similar approach, the skill developed in our experimental task can be modeled as a hidden Markov process. The analysis of the experimental data indicates that the skill, as quantified by the kinematic features, depends on the available sensory information and is adapted as the task is repeated. The samples of the velocity curve acquired during the experiment can be considered as observable symbols, with different states that model the velocity as the trial progressed, from the movement onset until reaching the contact position. In this way the course of velocity can be modeled as a *n* state left-right HMM. This trained HMM would represent a prototypical execution of the task, which can be queried by the robot in order to reproduce the movement. Based on the course of the prototypical execution, the robot can calculate the desired end effector variables in order to track a target path and to adjust, where necessary, the trajectory parameters (e.g., motion time, maximum velocity, and acceleration) according to its kinematic constraints (i.e., maximum joint velocities and maximum joint accelerations).

**Figure 14:**
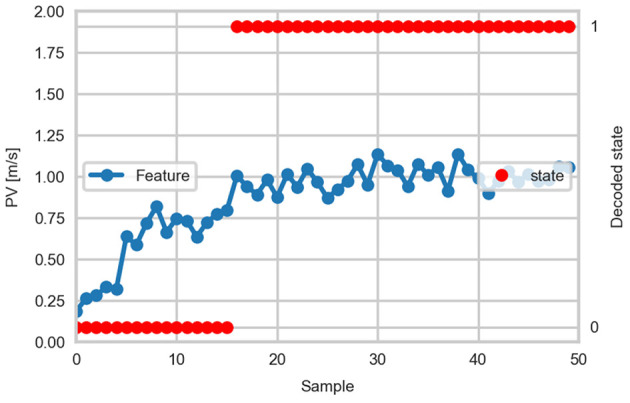
Example of the decoded state from a 2-state HMM learned from peak velocity data. One sample corresponds to one trial. The decoded state labeled as 0 corresponds to the learning phase and the state labeled as 1 corresponds to the phase of stable performance.

In addition to the modeling of the prototypical task execution, we propose to use a HMM in order to model the course of the adaptation of the kinematic parameters. In this case, the kinematic features observed throughout the experiment can be considered as observable symbols. Different states would model the parameters used by the person on a trial-to-trial basis and the state transitions would reflect the active perception strategy followed to accomplish the task. Such a model would be able to model the adjustment of the kinematic parameters (e.g., PV) from the low magnitudes observed during the first blocks of trials until achieving a stable magnitude as, for example, modeled with the asymptote of the exponential curve fit. Once the model has been obtained from the set of training data, the source of the adaptation process can be simulated. To exemplify this concept, a 2-state HMM was trained from the PV data from all participants, one state modeling the learning phase and another modeling the phase of stable performance. Ten training examples from the PV data across trials in the A condition (one example per participant) were used to learn the model parameters. Figure 14 shows the decoded states from the PV data of one participant.

The parameters of the fitted exponential curves and the HMMs can be used to identify the point in which performance reaches a stable point of operation. This enables the classification of trials as “learning” or “regular,” providing an interpretation of the variation of the observed performance as more repetitions are executed. This classification can be used to label a set of human demonstrations, which can be queried by the robotic system. Annotating the trials can help to reduce the search space when looking for a particular type of task execution. Thus, with the proposed approach it is possible to model performance during the learning phase or at a stage at which the person has achieved a stable performance, enabling the transfer of skill acquisition strategies and performance skills to robotic systems.

## 6. Conclusions

In this paper we have investigated how humans can use different sources of information to adapt their kinematic parameters in tool use. Specifically, we have measured the influence of haptic and auditory information in a virtual reality experiment, where the task was to learn how to move a virtual spoon with the hand in such a way that the contact velocity with an object assumes a specified level. Our data suggest the following conclusions: First, auditory information alone is basically sufficient for the learning of the movement of a tool toward an object with a specified contact force/velocity. It can be assumed that this is a general result, which is also valid in other situations, for example when a glass has to placed on a table without allowing for so much contact force that it will be shattered. Second, if haptic information is available, subjects learn faster, and act more precise, but they keep the contact force systematically reduced. This could be interpreted as a risk-avoidance strategy which ensures that the desired contact force is in no case substantially exceeded by the unavoidable, system-inherent variability of the movements, which could otherwise cause destruction.

In general, kinematics were adjusted according to the sensory information available to the motor control system. Furthermore, if multisensory information is available, the haptic information dominates. This is observed not only with respect to learning rate and precision, but also for the risk-avoiding systematic reduction of the average contact force level.

The differences in kinematic features across blocks of trials illustrate the course of adaptation as the task was repeated. The adaptation that results from the different active perception strategies can be modeled as a stochastic process. Two types of models can be obtained by means of HMMs from a set of realizations of a motor task: a prototypical task execution and the course of adaptation of the kinematic parameters, which enables the identification and labeling of different task executions as learning or regular trials. Both can be used as sources of information which can be queried by a robot in order to plan the motion of an end effector. It is still open for discussion which kinematic parameters may serve best as features for the training of a HMM. It may depend on the task, the constraints of the system (e.g. the robot's maximum acceleration) or the specifics of the movement's course, since some kinematic features carry more information on particular movements phases than others.

As shown in the related work section, acoustic information has been mainly used for the interactive recognition of household objects. In that context, the robot learns behavior-grounded object recognition models (Sinapov et al., 2011). In a similar fashion robots can learn behavior-grounded action models to perform manipulation tasks in which action parameters are matched to the sound they produce, achieving different levels of precision and accuracy. Thus, using acoustic information for the learning and adaptation of kinematic properties is possible and can be applied in robotics.

Behavior-grounded models for new applications need to consider the strategies used by humans to integrate the available sensory information and how we compensate the missing information. A central question is how auditory and haptic information modulate the kinematic parameters during a manipulation task. In order to extend the use of acoustic information into new applications, the behavior-grounded models must consider the reliability of this information source (as it might be affected by ambient or mechanical noise) and how humans cope with various levels of reliability. As reviewed in Bohg et al. (2017), research in interactive perception is mostly concerned with visual information, thus it is necessary to extend research toward a multi-modal framework. The experimental results show how different sensor modalities have an effect on the execution of a motor task. This paper provides preliminary evidence regarding the role of acoustic information in the learning and adaptation of kinematic properties and points at potential fields of application in robotics.

## Data Availability

The raw data supporting the conclusions of this manuscript will be made available by the authors, without undue reservation, to any qualified researcher.

## Ethics Statement

Ethics committee of the University of Bremen. All subjects gave written informed consent in accordance with the Declaration of Helsinki.

## Author Contributions

JM, TK, and CZ contributed conception and design of the experiment and wrote sections of the manuscript. JM programmed the virtual reality setup and contributed with analysis tools. JM and TK performed the experiment and wrote the first draft of the manuscript. TK performed the statistical analysis. All authors contributed to manuscript revision, read, and approved the submitted version.

### Conflict of Interest Statement

The authors declare that the research was conducted in the absence of any commercial or financial relationships that could be construed as a potential conflict of interest.
